# Leveraging patient-reported outcomes and serum markers to predict active ulcerative colitis: a multicenter cohort

**DOI:** 10.3389/fmed.2026.1755028

**Published:** 2026-04-09

**Authors:** Wenqian Li, Xin Hao, Yunyun Sun, Caimin Li, Mei Wang, Sicong Hou

**Affiliations:** 1 Department of Gastroenterology, The Affiliated Hospital of Yangzhou University, Yangzhou University, Yangzhou, Jiangsu, China; 2 Department of Gastroenterology, Baoying People’s Hospital, Yangzhou, Jiangsu, China; 3 Kangda College of Nanjing Medical University, Lianyungang, Jiangsu, China; 4 The First School of Clinical Medicine, Faculty of Medicine, Yangzhou University, Yangzhou, China; 5 Key Laboratory of the Jiangsu Higher Education Institutions for Integrated Traditional Chinese and Western Medicine in Senile Diseases Control, Yangzhou University, Yangzhou, China

**Keywords:** endoscopic activities, patient-reported outcomes, predictive model, serological indicators, ulcerative colitis

## Abstract

**Background and aim:**

This study aims to develop a score system via noninvasive and reliable clinical tools for individuals to distinguish remission and active Ulcerative Colitis (UC).

**Methods:**

We performed a retrospective multicenter study collecting 173 patients in the training cohort and 124 patients in the validation cohort with UC. Then we assessed the relationship between patient-reported outcomes (PROs) and serum indicators with endoscopic disease activity (defined as UCEIS ≥1). Univariate and multivariate logistic regression analyses were performed, with a stepwise backward selection approach used to select significant variables. Two predictive models were ultimately developed based on PROs and serum biomarkers. The performance of the models was evaluated through ROC curves, and calibration was assessed using Spiegelhalter’s Z-test.

**Results:**

A total of 173 and 124 patients were enrolled in the training and validation groups, respectively. Univariate and multivariate analyses revealed that stool frequency (SF), rectal bleeding (RB), CRP/TB, and PDW were significantly associated with endoscopic active UC. Two predictive models were developed, with SF (model A) and RB (model B) combined with CRP/TB and PDW, respectively. Both models demonstrated excellent discriminative ability for endoscopic activity, with area under the ROC curve (AUC) values of 0.906 (95% CI 0.863–0.949) and 0.899 (95% CI 0.855–0.943) in the training cohort. In the external validation cohort, the AUC values were 0.793 and 0.794, showing similar strong discriminative ability. In the Mayo Endoscopic subscore (MES) system, model A and model B exhibited AUC values of 0.894 and 0.884 in the training cohort, and 0.769 and 0.758 in the validation cohort. Subgroup analysis based on disease severity further validated the models’ stability and reliability.

**Conclusion:**

The predictive models based on SF and RB developed in this study demonstrated good discriminative ability in predicting endoscopic activity in patients with UC. Both models performed well in the internal and external validation. Additional validation utilizing the MES and disease severity provided further evidence supporting the reliability and effectiveness of these models. These findings underscored the potential clinical utility of the SF- and RB-based models as valuable tools for predicting endoscopic disease activity in UC patients, which could facilitate more informed clinical decision-making and improve patient outcomes in the management of UC.

## 1 Introduction

Ulcerative colitis (UC) is a chronic, idiopathic inflammatory disorder primarily affecting the colonic mucosa, with hallmark clinical manifestations including recurrent abdominal pain and bloody diarrhea. Over the past three decades, the age-standardized incidence rate of IBD in China has increased by 89.19%, and the age-standardized prevalence rate making a 63.86% rise (1, 2). A nearly 15-year hospital cohort study in China demonstrated that approximately 58% of patients experienced a disease course marked by recurrence or persistent activity (3). Likewise, a systematic review highlighted that among patients who achieved remission following treatment, the recurrence rate within 1 year could reach 25.4 to 37.4% (4). Consequently, precise and timely monitoring of disease activity is essential to prevent disease progression and guide treatment.

Endoscopy, an indispensable tool for monitoring UC progression, enables direct visualization of mucosal surfaces and allows precise evaluation of inflammatory burden, including distribution, severity, and characteristic lesions (e.g., loss of vascular texture, erosions, or ulcerations) (5, 6). This may provide clinicians with the most objective imaging evidence for therapeutic decision-making. Crucially, clinical trials have shown that endoscopic mucosal remission is associated with improved long-term prognoses in UC, including reduced hospitalization frequency and lower colectomy rates (7, 8). Therefore, endoscopy not only plays a pivotal role in assessing disease activity but also serves as a key criterion in guiding the establishment of treatment goals (9–11). The Ulcerative Colitis Endoscopic Index of Severity (UCEIS) and the Mayo Endoscopic subscore (MES) are reliable instruments for measuring the endoscopic disease activity of UC. Generally, MES = 0 or UCEIS = 0 is considered as the remission state of UC (12). Nevertheless, since endoscopy is invasive and expensive and patients will need frequent utilization of endoscopy in their lifetimes, the application of endoscopy might be limited in long-term follow-up. Against this backdrop, an non-invasive and convenient prediction method for monitoring disease progression urgently needs to be developed.

Patient-reported outcomes (PROs), which directly capture patients’ subjective symptoms, included rectal bleeding (RB), stool frequency (SF) and diarrhea, represent the most immediate metric in terms of quality-of-life (13, 14). In a prospective, cross-sectional cohort study, RB (*ρ* = 0.53, *p* < 0.001; ρ = 0.52, *p* < 0.001) and SF (ρ = 0.44, *p* < 0.001; ρ = 0.43, *p* < 0.001) were both significantly correlated with the MES and UCEIS (15). These results indicate that PROs can effectively reflect disease activity, their measurement can not only empower patients to better manage their conditions at home, but also provide clinicians with real-time information on the disease state, which is particularly significant in primary healthcare settings. Moreover, a multi-center study carried out by Colombel et al. demonstrated that the sensitivities and specificities of RB, SF and RB + SF for detecting mucosal remission were 77 and 81%, 62 and 95%, and 54 and 95%, respectively (13). Evidently, PRO demonstrates limited sensitivity in predicting disease remission, which may result in either underestimation or overestimation of disease activity. For one thing, underestimation could delay timely therapeutic intervention, thereby increasing the risk of complications and progression to refractory disease states. Conversely, overestimation may prompt unnecessary treatment intensification, potentially exposing patients to drug-related adverse effects.

To overcome this, the exploration of serological indicators that are more consistently with endoscopy-defined disease activity has emerged as a new direction in clinical research. Inflammatory markers such as C-reactive protein (CRP), erythrocyte sedimentation rate (ESR), and fecal calprotectin (FC), as well as pro-inflammatory cytokines including interleukin 6 (IL-6), IL-8, and tumor necrosis factor-alpha (TNF-*α*), exhibit good correlation with endoscopic disease activity scores. To further avoid interference from systemic inflammation and medications, researchers are actively investigating multi-marker predictive models to enable a more comprehensive assessment of disease activity. For instance, CRP/ALB can effectively distinguish between active and remission phases of UC (AUC = 0.853, sensitivity = 76.8%, specificity 84.8%) (16). Additionally, a combination biomarker for predicting endoscopic activity was proposed, consisting of CRP, neutrophil, lymphocyte, which achieved an AUC = 0.788 (sensitivity = 77.0%, specificity = 59.0%) (17). Despite making great efforts, to our knowledge, no ideal predictive indicator is an alternative to endoscopy in daily clinical practice.

In the current study, we using baseline PROs and easily accessible blood indicators (including CRP, TB and PDW) to establish predictive models, and these models have been robustly validated against established standards, including the Mayo score and disease severity based on UCEIS, aiming to assist patients dynamically monitor their own disease activity and assist primary medical institutions in managing patients.

## 2 Methods

### 2.1 Study design and patients

This retrospective multicenter cohort study consisted of 297 UC patients who were admitted in the electronic medical database of two tertiary hospitals between January 2017 to March 2024. The training cohort including 173 patients was enrolled from Affiliated Hospital of Yangzhou University, and external validation cohort including 124 patients was recruited from Baoying People’s Hospital. The discharge diagnosis of “ulcerative colitis” was the main focus of the search terms. The inclusion criteria were: (1) Diagnosis of UC based on clinical consensus (18); (2) Patients available for entire colonoscopy. (3) Data from the laboratory examination and endoscopy must be accessible at the same time, and the laboratory examination findings must have been received no more than 7 days prior or following the colonoscopy which including biomarkers such as blood cell counts, protein nutrition; Exclusion criteria included: (1) Missing clinical data; (2) Bowel resection or incomplete bowel examination; (3) Coexistence of ankylosing spondylitis, Sjogren’s syndrome, systemic lupus erythematosus; (4) Malignant tumor, hematological diseases, serious infection, or impaired liver infections affect the results of laboratory tests; and (5) Pregnancy or lactation. This study was approved by the Research Ethics Committee of the Affiliated Hospital of Yangzhou University and Baoying People’s Hospital.

### 2.2 Endoscopic data and outcomes

Experienced endoscopists retrospectively review the endoscopic photos and descriptions via the endoscopic image reporting system, taking into account factors such as vascular pattern, bleeding, and erosion ulcers. Endoscopic scoring was carried out independently by two experienced endoscopists. In situations where their scores disagreed, the ultimate score was determined by discussing a consistent score. The endoscopists were blinded to the laboratory parameter values in order to prevent any bias. According to clinical guidelines, endoscopic activity is categorized as remission (UCEIS = 0 or MES = 0), and activity (UCEIS > 0 or MES > 0) (19, 20). We use UCEIS scoring system for the purpose of model construction while MES for validation.

### 2.3 Clinical data collection

Data obtained from our center’s electronic medical database, age, gender, temperature, pulse, SF, hematochezia, duration, extent of disease, blood indicators: Lymphocyte count (L, *10^9^/L), Platelet count (PLT, *10^9^/L), Red blood distribution width (RDW, fl), Red blood cell (RBC, *10^12^/L), Hemoglobin (Hb, g/L), Platelet distribution width (PDW, fl), Total bilirubin (TB, umol/L), Albumin (ALB, g/L), C-reactive protein (CRP, mg/L), Erythrocyte sedimentation rate (ESR, mm/h).

### 2.4 Statistical analysis

Categorical variables were presented in terms of frequency and percentage, and groups were compared using Chi-square tests or Fisher’s exact tests. Kolmogorov–Smirnov tests were utilized to check the normality of continuous variables, while the independent samples *t*-test was employed for comparison and the mean and standard deviation (SD) were utilized for normally distributed data. When comparing non-normally distributed variables, the Mann–Whitney U tests were employed to determine the median and interquartile range (IQR).

The study employed logistic regression analysis to evaluate the correlation between non-invasive indicators and the possibility of endoscopic activity, defined by an endoscopic UCEIS score of ≥1. Collinearity diagnostics were conducted to mitigate risks of model overfitting, and a stepwise backward selection approach was applied in the multivariate analysis to retain variables with a univariate significance of *p* < 0.05. The coefficient estimates derived from the multivariate regression analysis provided a statistical basis for constructing the endoscopic activity predictive score.

Both the discrimination and calibration abilities were used to evaluate the predictive performance: the discrimination was evaluated using the consistency index (C-index), the area under the receiver operating characteristic curve (AUROC). The Spiegelhalter Z-test was used to evaluate the fit of the model. The validation of the model included both internal and external validation, handled by the modeling cohort and the validation cohort, respectively. To determine the performance of the calibration and discrimination, internal validation was carried out using the bootstrap approach at 1000 repeats. Two-tail *p* < 0.05 were taken to be statistically significant. All statistical analysis was performed by using SPSS (version 27.0, IBM Corporation, Chicago, United States) and R (version 4.3.0 R Foundation for Statistical Computing, Vienna, Austria). Graphpad Prism 8 was used for plotting.

## 3 Results

### 3.1 Patient characteristics

The study comprised a total of 297 UC patients, and all patients met the clinical consensus criteria for UC diagnosis. Table 1 depicts the demographic and clinical characteristics of the recruited patients. A total of 173 patients (97 males and 76 females; median age: 49 years; IQR: 38–62 years) were included in the training cohort from January 2017 to March 2024; 124 patients in the external validation cohort (66 males and 58 females; median age: 52 years; IQR: 42–59 years). According to the MES, the activity rates in training and validation cohort were 73.41% and 68.55%, respectively. Based on the UCEIS, the activity rates of the two cohorts were comparable (69.36% vs 69.35%). Among patients with active disease, the proportions of mild activity were 45.66% and 48.39%, and the proportions of moderate-to-severe activity were 23.70% and 20.97% in training and validation cohort, respectively. (21). In both cohorts, 5-aminosalicylic acid (5-ASA) was the primary treatment agent for the patients, with a minor percentage of patients receiving biologics for escalating therapy.

**Table 1:** Demographic and clinical characteristics of the enrolled UC patients.

Variables	Training cohort (*n* = 173)	Validation group (*n* = 124)	*P*
Gender, *n* (%)			0.627^b^
Male	97 (56.07)	66 (53.23)	
Female	76 (43.93)	58 (46.77)	
Temperature (°C)	36.60 (36.50, 36.70)	36.70 (36.58, 36.80)	0.116^c^
Pulse (times/min)	71.00 (67.00, 78.00)	72.00 (68.00, 74.00)	0.490^c^
Age (years)	49.00 (38.00, 62.00)	52.00 (42.00, 59.00)	0.363^c^
Duration of disease (mouths)	39.00 (9.00, 84.00)	37.50 (24.00, 70.50)	0.283^c^
MES			0.361^b^
Remission	46 (26.59)	39 (31.45)	
Activity	127 (73.41)	85 (68.55)	
UCEIS			0.999^b^
Remission	53 (30.64)	38 (30.65)	
Activity^a^	120 (69.36)	86 (69.35)	
Mild	79 (45.66)	60 (48.39)	
Moderate-to-severe	41 (23.70)	26 (20.97)	
Lymphocyte (10^9^)	1.60 (1.20, 2.12)	1.63 (1.33, 2.03)	0.408^c^
PLT (10^9^)	213.00 (178.00, 266.00)	219.50 (172.75, 285.25)	0.556^c^
PDW (fl)	14.90 (12.20, 16.20)	14.30( (11.90, 16.91)	0.832^c^
RBC (10^12^)	4.38 (3.95, 4.90)	4.425 (3.93, 4.84)	0.966^c^
Hb (g/L)	130.00 (117.00, 147.00)	128.50 (114.00, 140.75)	0.104^c^
RDW (%)	12.70 (12.30, 13.30)	13.37 (12.10, 14.67)	0.062^c^
CRP/TB	2.33 (0.52, 16.07)	2.76 (0.69, 8.17)	0.698^c^
TB (umol/L)	10.10 (3.80, 30.70)	11.36 (5.42, 16.42)	0.410^c^
ALB (g/L)	40.30 (35.90, 44.70)	43.20 (34.80, 46.50)	0.162^c^
CRP (mg/L)	29.38 (10.00, 94.26)	27.36 (4.77, 73.95)	0.248^c^
ESR (mm/h)	14.00 (7.00, 25.00)	16.50 (10.00, 32.75)	0.101^c^
RB	1.00 (1.00, 2.00)	2.00 (1.00, 3.00)	0.062^c^
SF	1.00 (1.00, 1.67)	2.00 (1.00, 2.00)	0.131^c^
Medication			0.122^b^
5-ASA	134	105	
Biologics	39	19	

Continuous variables were expressed as mean ± standard deviation or median [interquartile range (IQR)]. Categorical variables were expressed as frequencies (percentages). UC, ulcerative colitis; PLT, platelet; PDW, platelet distribution width; RBC, red blood cell; Hb, hemoglobin; RDW, red blood cell distribution width; CRP, C-reactive protein; TB, total bilirubin; ALB, albumin; ESR, erythrocyte sedimentation rate; RB, rectal bleeding; SF, stool frequency; 5-ASA, 5-aminosalicylic acid.

a UC activity were classified into mild (UCEIS < 4) and moderate-to-severe (UCEIS ≥ 4).

b Chi-square test, *p* < 0.05 was considered statistically significant.

c Mann–Whitney test, *p* < 0.05 was considered statistically significant.

### 3.2 Prediction model construction

Univariate analysis showed higher lymphocyte, PDW, and albumin were associated with a lower probability of active disease, while elevated CRP/TB, ESR, RB, and SF were associated with an increased risk of active disease (Table 2). The above-mentioned variables were evaluated in a multivariable logistic regression using a backward stepwise regression method when collinearity was taken into account (Table 3), turning out two different combinations involving SF, RB, CRP/TB, PDW. According to the estimates of regression coefficients, a prediction model for endoscopic activity was established. The model A total score = 1.462 * SF + 0.597 * CRP/TB − 0.139 * PDW. The model B total score = 1.551 * RB + 0.516 * CRP/TB − 0.155 * PDW.

**Table 2:** Univariate analysis on variables associated with possibility of active disease (UCEIS >0; Mayo >0) in patients with UC.

Variables	*P*	OR	95%CI
Lymphocyte	0.063	0.60	0.36 ~ 0.99
PLT	0.137	1.00	1.00 ~ 1.01
PDW	0.003	0.83	0.74 ~ 0.94
WBC	0.981	1.00	0.71 ~ 1.39
Hb (g/L)	0.196	0.99	0.97 ~ 1.01
RDW (%)	0.292	1.15	0.89 ~ 1.49
CRP/TB	<0.001	1.74	1.31 ~ 2.33
ALB	0.002	0.92	0.88 ~ 0.97
ESR	0.003	1.04	1.01 ~ 1.07
RB	<0.001	5.87	2.86 ~ 12.07
SF	<0.001	4.19	2.38 ~ 7.38

**Table 3:** Multivariable analysis on the possibility of active disease (UCEIS >0; Mayo >0) in patients with UC.

Variables	*P*	OR	95%CI
PDW	0.040	0.823	0.684 ~ 0.992
CRP/TB	0.002	1.712	1.209 ~ 2.423
RB	<0.001	5.092	2.033 ~ 12.754
SF	<0.001	4.207	1.921 ~ 9.213

**Figure 1:**
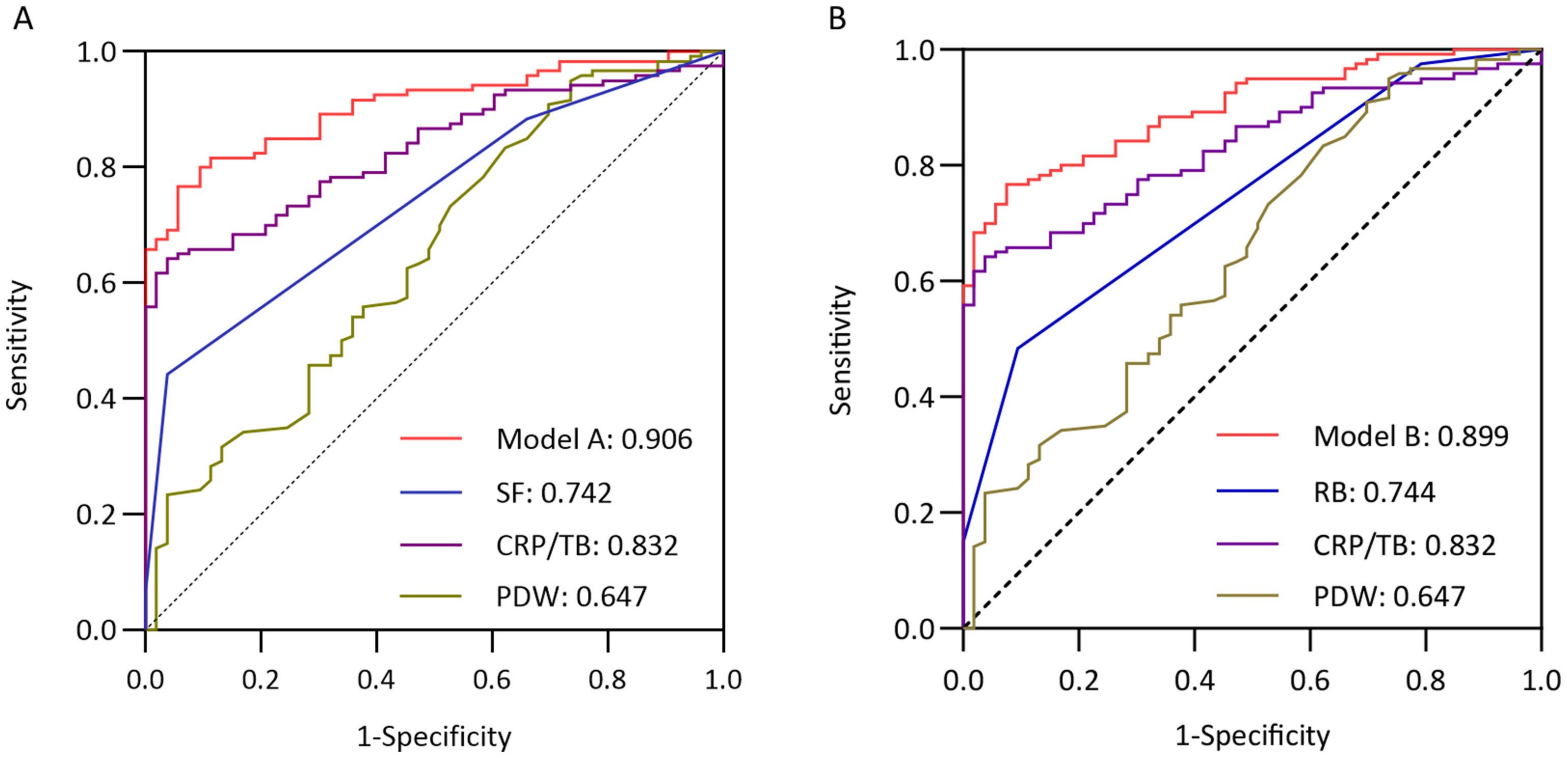
Receiver operating characteristic (ROC) curves for two predicted models for possibility of active UC in training cohort. **(A)** Model A showed a good discrimination with area under curve (AUC) of 0.906 [95% confidence interval (CI) 0.863–0.949]. The sensitivity and specificity under the cut-off value of 0.72 performed were 76.7 and 94.3%, respectively. **(B)** Model B showed a good discrimination with AUC of 0.899 (95% CI 0.855–0.943). The sensitivity and specificity under the cut-off value of 0.676 performed were 76.7 and 92.5%, respectively.

For the two models predicting endoscopic activity, receiver operating characteristic (ROC) curves demonstrated strong discriminatory ability for identifying endoscopic activity, with areas under the curve (AUC) of 0.906 [95% confidence interval (CI) 0.863–0.949, *p* < 0.001] and 0.899 (95% CI 0.855–0.943, *p* < 0.001), respectively (Figures 1A,B). In Model A, using a cut-off value of 0.72, the sensitivity for distinguishing active endoscopic disease from remission was 76.7%, and the specificity was 94.3%. In addition, 0.676 was regarded as cut-off value in model B under which sensitivity and specificity were 76.7 and 92.5%.

**Figure 2:**
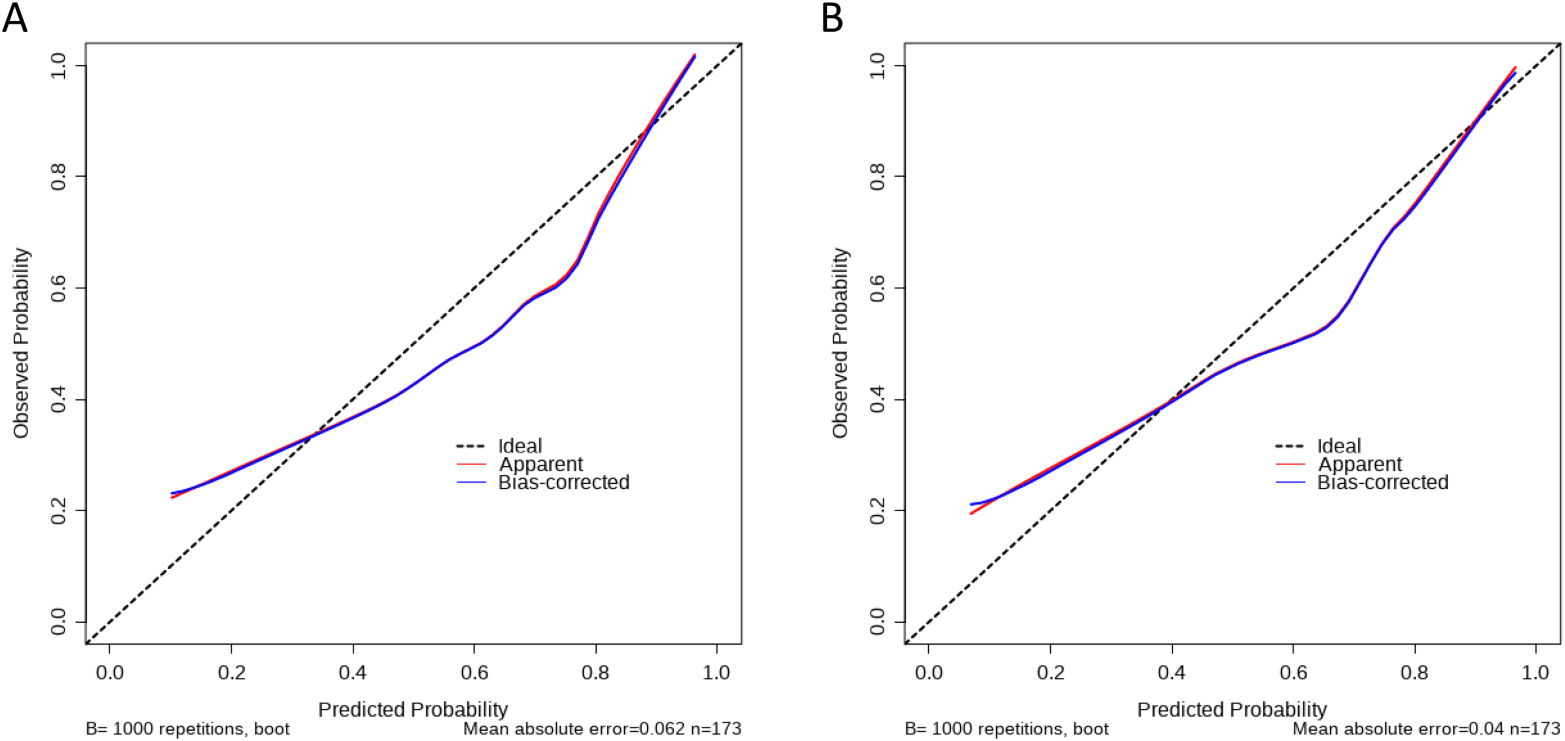
Calibration curves of two models for the prediction of activity status in the training group. **(A)** Calibration curve of model A based on SF, CRT/TB, and PDW. **(B)** Calibration curve of model B based on RB, CRT/TB, and PDW. The black dot line on the diagonal of the figure indicated a complete fitting between predictive model and actual data. The red solid line and black solid line illustrated the degree of fitness between model prediction and actual probability of activity UC.

In terms of calibration, the two prediction models showed good fit using the Spiegelhalter Z-test (*p* = 0.159 in model A and *p* = 0.171 in model B). The calibration curves showed an acceptable level of agreement between the predicted probabilities and the actual proportions of endoscopic active UC (Figures 2A,B).

### 3.3 External evaluation of the prediction models’ performance for activity UC

**Figure 3:**
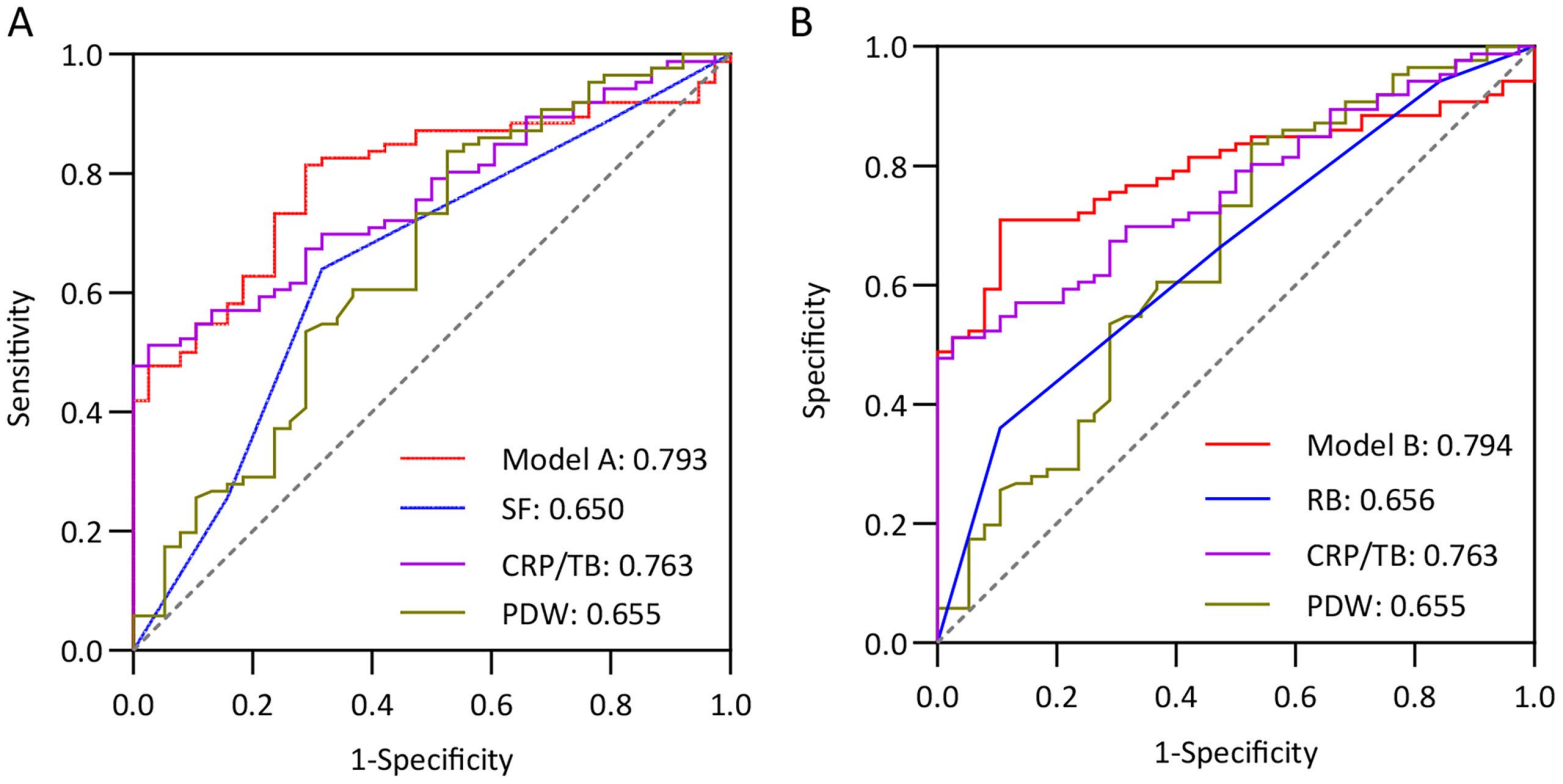
ROC curves for two predicted models for possibility of activity UC in validation group. **(A)** Model A showed a good discrimination with area under curve (AUC) of 0.793 (95% CI 0.714–0.872). The sensitivity and specificity under the cut-off value of 0.761 performed were 87.2% and 52.6%, respectively. **(B)** Model B showed a good discrimination with AUC of 0.794 (95% CI 0.717–0.872). The sensitivity and specificity under the cut-off value of 0.987 performed were 83.7% and 50.0%, respectively.

**Figure 4:**
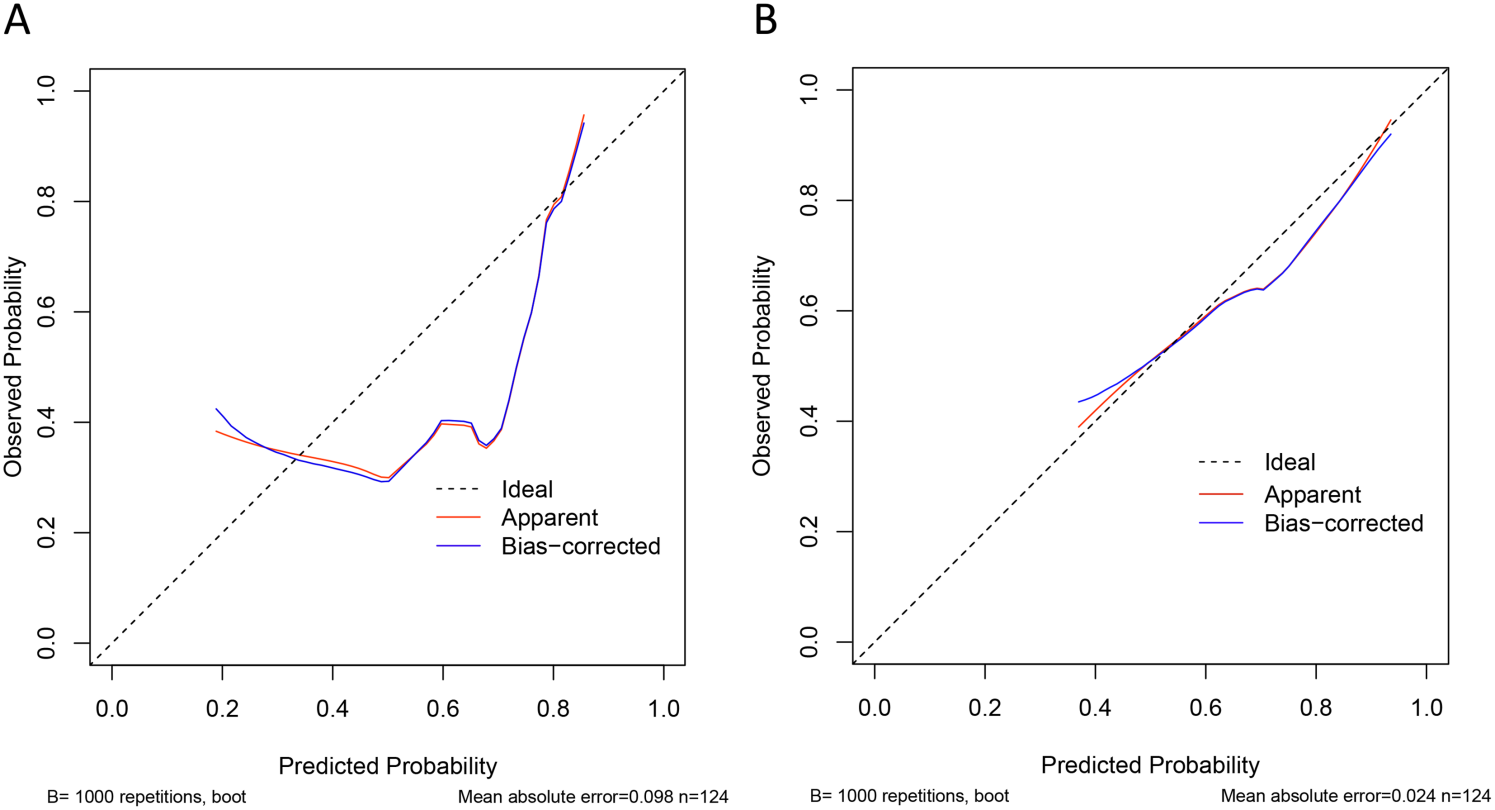
Calibration curves of two models for the prediction of activity UC in the validation group. **(A)** Calibration curve of model A based on SF, CRT/TB, and PDW in validation group. **(B)** Calibration curve of model B based on RB, CRT/TB, and PDW in validation group. The black dot line on the diagonal of the figure indicated a complete fitting between predictive model and actual data. The red solid line and black solid line illustrated the degree of fitness between model prediction and actual probability of activity UC.

Meanwhile, we further validated the discriminatory ability of the models in the external validation cohort, which also showed excellent performance. The AUC of model A and model B in external cohort was 0.793 (95% CI 0.714–0.872) and 0.794 (95% CI 0.717–0.872), respectively (Figures 3A,B). From calibration analysis, both models had a relatively consistent tendency with the ideal condition (Figures 4A,B).

### 3.4 Evaluation of the prediction models’ performance based on MES

**Figure 5:**
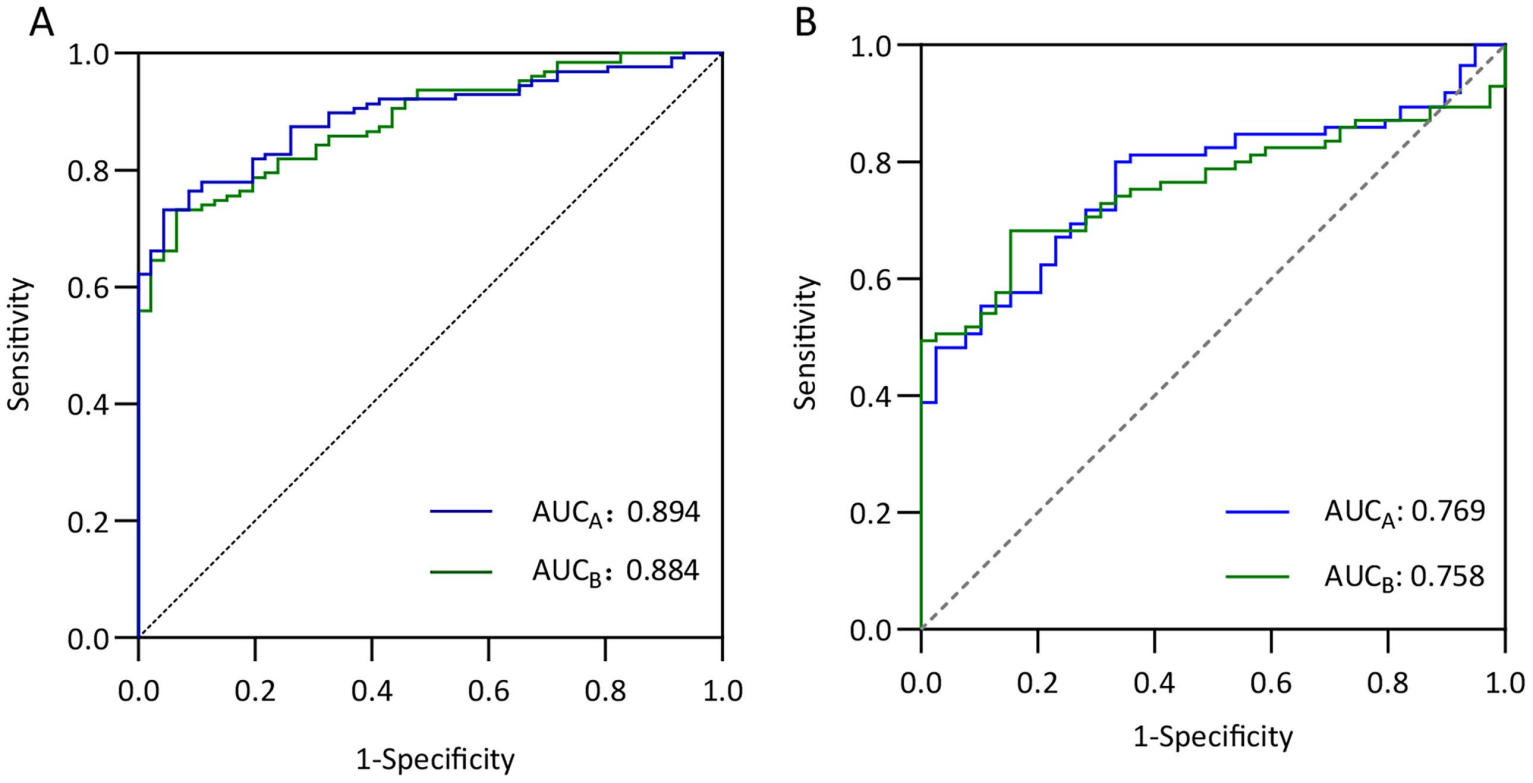
Evaluation of the prediction models’ performance based on MES. **(A)** ROC curves of Model A (AUC = 0.894, 95% CI 0.847–0.939) and Model B (AUC = 0.884, 95% CI 0.835–0.932) in the training cohort. **(B)** ROC curves of Model A (AUC = 0.769, 95% CI 0.688-0.851) and Model B (AUC = 0.758, 95% CI 0.676–0.840) in the external validation cohort.

To comprehensively evaluate the predictive performance of the two models, we chose to use the MES as the validation criterion, and MES > 0 was defined as disease activity. By comparing the models’ total scores with the MES scores, we evaluated the models’ predictive ability within the MES system. As shown in the Figure 5A, in the training cohort, both Model A and Model B demonstrated excellent discriminatory efficacy, with AUC of 0.769 (95% CI 0.688–0.851) and 0.758 (95% CI 0.676–0.840), respectively. In the external validation cohort (Figure 5B), the AUC values of Model A and Model B reached 0.769 (95% CI 0.688–0.851) and 0.758 (95% CI 0.676–0.840), respectively. These fully verified the generalization ability of the models in different endoscopic scoring systems.

### 3.5 Evaluation of the prediction models’ performance based on disease severity

To further validate the performance of the models, patients were stratified according to the UCEIS score, with UCEIS < 4 defined as mild disease activity, and UCEIS ≥4 defined as moderate-to-severe activity. The discriminative ability of the models was subsequently evaluated.

**Figure 6:**
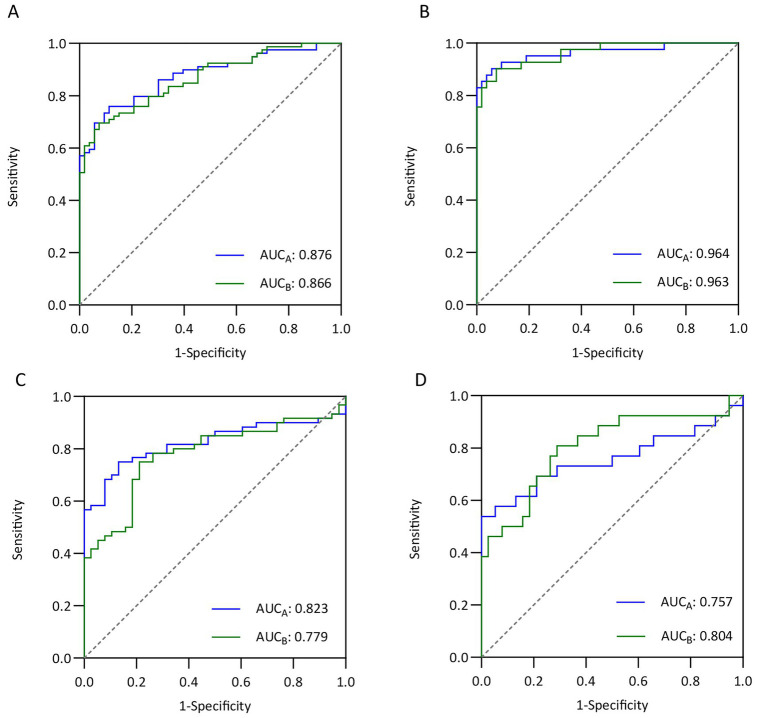
Evaluation of the prediction models' performance in distinguishing disease activity from remission. **(A)** ROC curves in the training cohort for distinguishing mild activity from remission. **(B)** ROC curves in the training cohort for distinguishing moderate-to-severe activity from remission. **(C)** ROC curves in the validation cohort for distinguishing mild activity from remission. **(D)** ROC curves in the validation cohort distinguishing moderate-to-severe activity from remission.

In the training cohort, both models demonstrated good discriminative ability. For differentiating mild activity from remission, Model A achieved an AUC of 0.876 (95% CI 0.818−0.933), and Model B achieved an AUC of 0.866 (95% CI 0.807−0.925) (Figure 6A). For differentiating moderate-to-severe activity from remission, the discriminative performance further improved, with AUCs of 0.964 (95% CI 0.924−1.000) for Model A and 0.963 (95% CI 0.928−0.997) for Model B (Figure 6B). In the validation cohort, the models maintained acceptable predictive performance. For mild activity versus remission, the AUC were 0.823 (95% CI 0.739−0.907) for Model A and 0.779 (95% CI 0.686−0.871) for Model B (Figure 6C). For moderate-to-severe activity versus remission, the AUC were 0.757 (95% CI 0.619−0.895) and 0.804 (95% CI 0.687−0.920) for Model A and Model B, respectively (Figure 6D). Overall, both models showed stable discriminative ability in the training and validation cohorts.

In conclusion, the results of the subgroup analysis consistently indicate that the prediction model developed in this study can be applied to patients with UC of different disease severity, showing crucial clinical implications for identifying high-risk patients and guiding intensive treatment.

## 4 Discussion

This study established predictive models for endoscopic active UC by integrating clinical and biochemical parameters. We identified four robust predictors of active UC: RB, SF, CRP/TB, and PDW, and developed two distinct models incorporating these variables. Both models effectively compensate for the limitations of single-parameter assessments of active disease, offering both non-invasive characteristics and high clinical utility in routine practice.

The Selecting Therapeutic Targets in Inflammatory Bowel Disease (STRIDE) committee recommended that PROs, such as resolution of RB and bowel habit normalization, should be a therapeutic target for UC (22). Actually, disease control based on symptoms alone is not sufficient because the manifestation of symptoms may lag behind the development of mucosal injury or inflammatory responses (23). The facts that approximately 70% of IBD patients in clinical remission show inflammation during endoscopy, and 30–50% of IBD patients have irritable bowel syndrome (IBS) like symptoms without having mucosal inflammation during endoscopy underscored this (24, 25). Our model integrates SF and RB with PDW and CRP/TB, respectively, establishing a novel multi-dimensional fusion approach that combines symptomatic data with blood biomarkers, which can provide quantitative support for determining whether patients are in a state of remission or active disease. More importantly, our model offers valuable insights for formulating symptom-guided therapeutic strategies. Specifically, a high score in the “SF model” indicates potential intestinal motility disorders or bile acid metabolic abnormalities (26–28). Intervention priorities should thus focus on regulating intestinal peristalsis, enhancing bile acid reabsorption, and increasing dietary fiber intake to prolong intestinal transit time. Secondly, an elevated score in the “RB model” strongly suggests inadequate mucosal healing or persistent mucosal inflammation (29, 30), warranting the timely initiation of biological agents.

In this study, univariate and multivariate analyses were conducted to investigate the potential associations between various factors and UC activity. Statistical results indicated that both PDW and CRP/TB were significantly correlated with active UC, which were consistent with previous studies. For instance, Shi et al. compared the subgroups of patients with different UC severities and then concluded that the TB level was significantly higher in the mild activity subgroup than in the moderate and severe activity subgroups (*p* < 0.05), which could be an independent protective factor for UC (*p* < 0.001, 95% CI 0.794–0.918) (31). Subsequently, A retrospective study demonstrated that the AUC for CRP/TB was 0.735, which was higher than the AUC values for either CRP (0.704) or TB (0.691) alone when determining endoscopic mucosal improvement (32). Based on the aforementioned results, it becomes evident that composite indicators possess distinct advantages in predicting disease activity. Regarding PDW, in a retrospective analysis enrolled 249 severely or moderately active UC patients and 50 healthy subjects, Gerçeker et al. observed a strong negative correlation between PDW levels and Mayo score (*r*=-0.805, *p* <0.001). (33). Moreover, a meta-analysis included 8,350 IBD patients revealed that the active UC group exhibited significantly lower PDW levels than the control group [weighted mean difference (WMD) = −1.138, 95% CI -1.535, −0.741%] (34). At the molecular level, bilirubin may alleviate intestinal damage through its potent antioxidant properties: scavenging free radicals and modulating redox reactions (35), while during inflammatory conditions, active platelets are more readily aggregate in blood to participate in thrombus formation, reducing platelet size heterogeneity in the peripheral circulation. Our further research demonstrated superior predictive performance of comprehensive models compared to a single symptom or serum indicator. Specifically, in the training cohort, the AUC of SF-based and RB-based model was 0.906 and 0.899, respectively, which were higher than that of SF (0.742), RB (0.744), CRP/TB (0.832), and PDW (0.647), accompanied by high sensitivity and specificity. Although our models’ performance is promising, further large-scale validation is essential to confirm its robustness and establish its clinical utility in diverse patient populations.

Our study has several limitations. First, although this study leveraged multi-center data for model development, the relatively limited sample size may increase the risk of overfitting, thereby compromising the models’ stability. To address this issue, future work should involve larger patient cohorts, which would help validate our results and enhance the robustness and clinical utility of the proposed model. Additionally, while we have developed a non-invasive, multi-dimensional evaluation system, PROs rely on patient self-reporting to a certain extent. For example, these outcomes are vulnerable to recall bias, as patients may not accurately remember the frequency or severity of their symptoms, especially over extended periods. Also, different patients may assess their symptoms in varying ways according to individual interpretation, which can be influenced by personal tolerance to discomfort, or cultural factors. This variability will introduce inconsistencies in the data, potentially affecting the accuracy of the model. Therefore, while PROs provide valuable insights into disease activity, their reliability and validity must be further assessed in prospective studies, and their integration with more objective markers should be refined to enhance model performance and predictive precision.

In summary, we developed validated predictive models for active UC using non-invasive indicators, including SF, RB, PDW, and CRP/TB, which provide a novel tool for assessing disease activity not only in comprehensive care settings but also in primary and community healthcare institutions, facilitating more accessible cost-effective disease management. The models were rigorously validated through internal, external, and different disease assessment system, and disease severity-based subgroup analysis, showing strong consistency between predicted probabilities and actual outcomes. Although emerging biomarkers, such as molecular markers, immunological markers, microbiome data, and imaging data, hold significant potential for improving disease prediction, their widespread adoption is still limited due to challenges in standardization and clinical implementation. Future research could focus on integrating these biomarkers into predictive models, with particular attention to their ability to capture the multi-dimensional and dynamic nature of UC, enabling more personalized, timely, and precise interventions for IBD patients across various healthcare levels.

## Data Availability

The raw data supporting the conclusions of this article will be made available by the authors, without undue reservation.
